# Supplementary material for: MTCH2-mediated mitochondrial fusion drives exit from naïve pluripotency in embryonic stem cells
Source: Nat Commun. 2018 Dec 3;9:5132. doi: 10.1038/s41467-018-07519-w (PMC6277412; doi:10.1038/s41467-018-07519-w)
Supplement: Supplementary file 1 — Supplementary Information [file 41467_2018_7519_MOESM1_ESM.pdf]

Supplementary information

MTCH2-mediated mitochondrial fusion drives exit from naïve pluripotency in embryonic stem cells

Bahat et al.

Supplementary figures

Supplementary figure 1

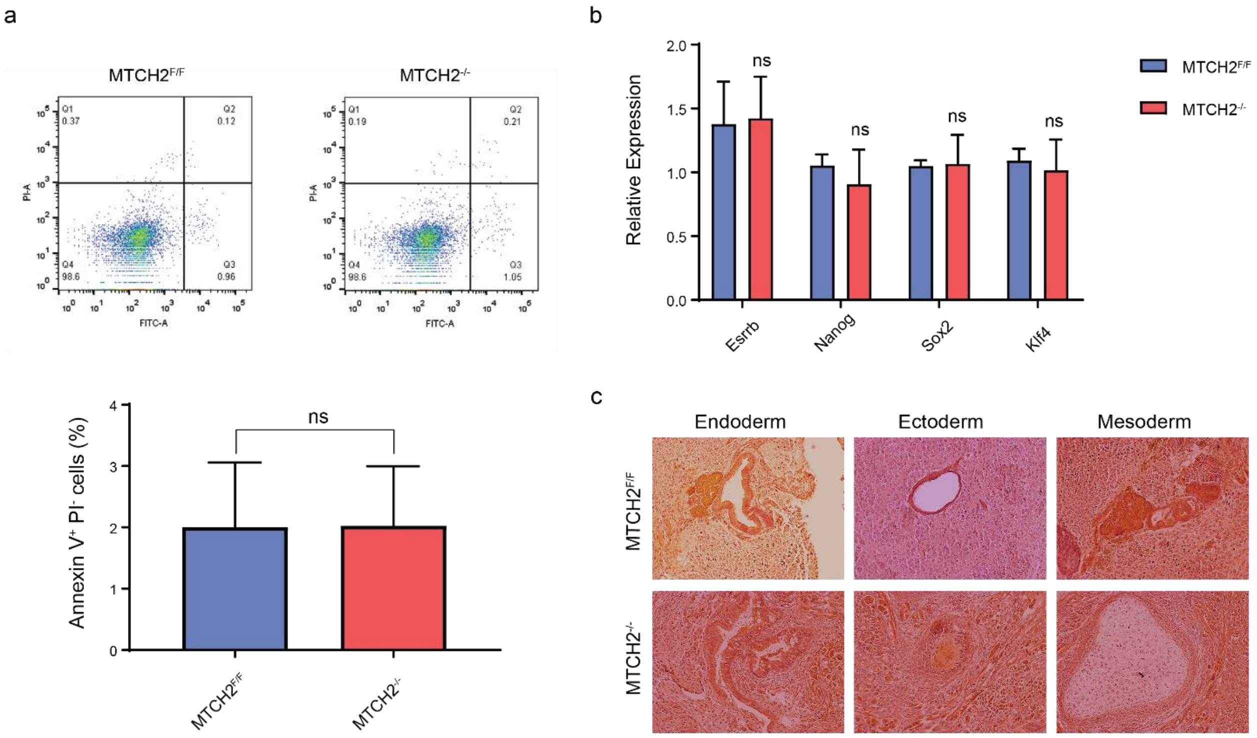

Supplementary figure 1. Loss of MTCH2 in ESCs does not impair naïve pluripotency. (A) *MTCH2$^{F/F}$* and *MTCH2$^{-/-}$* ESCs show low and similar levels of apoptosis. The levels of apoptosis were assessed by FACS analysis using propidium iodide (PI) and annexin V (FITC) staining's (Upper panels). Results are presented as mean ± SD (n=3) (Lower panel). **(B)** *MTCH2$^{-/-}$* ESCs maintain levels of core naïve pluripotency genes. *MTCH2$^{F/F}$* and *MTCH2$^{-/-}$* ESCs were analyzed for the levels of naïve pluripotency genes by qPCR. Results are presented as mean ± SD (n=3). **(C)** *MTCH2$^{-/-}$* ESCs maintain the ability to differentiate *in vivo*. H&E staining of paraffin sections reveals characteristic three germ layer structures in the *MTCH2$^{F/F}$* ESC-injected mice (Ciliated epithelia - endoderm, keratin – ectoderm and glandular structure - mesoderm) and in the *MTCH2$^{-/-}$* ESC-injected mice (Goblet cells - endoderm, keratin – ectoderm, cartilage - mesoderm).

Supplementary figure 2

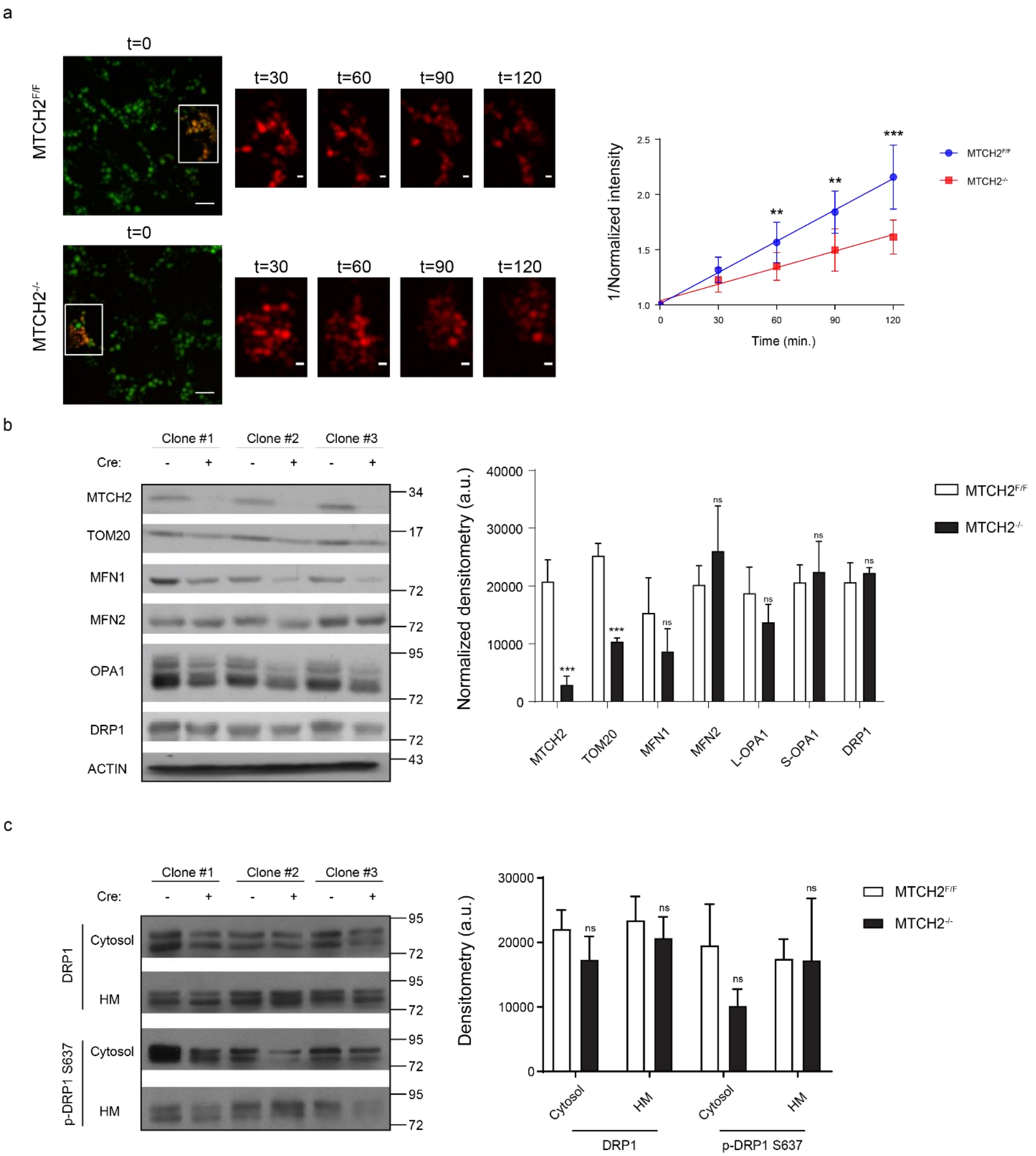

Supplementary figure 2. Loss of MTCH2 in ESCs decreases the rate of mitochondrial fusion. (A) *MTCH2*$^{-/-}$ ESC mitochondria possess a lower fusion rate. ESCs expressing a matrix targeted mito-dendra2 green fluorescent protein were laser-excited to photo-convert a subset of green

fluorescence mitochondria to red fluorescent mitochondria. Left and middle panels: mt-RFP fluorescence immediately after photoconversion (t=0; Insets in left panels, scale bar 5 μm) and 30, 60, 90 and 120 minutes after photoconversion (middle panels, scale bar 1 μm) in *MTCH2*$^{F/F}$ and *MTCH2*$^{-/-}$ ESCs (upper and lower panels, respectively). Right panel: quantification of mitochondrial fusion rate as 1/mt-RFP fluorescence within the initially photoconverted region throughout time. An average of three independent experiments is presented as mean ± SEM (**p<0.01, ***p<0.001; n=10). **(B)** Loss of MTCH2 does not alter the expression levels of the major mitochondrial dynamics regulators. Left panel: Western blot analysis of whole cell lysates prepared from three different *MTCH2*$^{F/F}$ ESC clones (clone #1, #2, #3) either left untreated (-) or treated with Cre recombinant *in vitro* (+). Right panel: densitometry analysis of the protein bands presented in the left panel normalized to the levels of TOM20 and Actin, used as a loading control (the averages of the bands from three clones are presented). Note that MTCH2 knockout results in a decrease the levels of TOM20, which may indicate on a decrease in mitochondrial mass. Results are presented as mean ± SD (***p<0.001; n=3). **(C)** Loss of MTCH2 does not alter the mitochondria/cytosolic levels of DRP1 and p-DRP1 S637. Left panel: Western blot analysis of heavy membrane (HM; mitochondria-enriched) and cytosolic fractions prepared from the three clones presented in (B). Right panel: densitometry analysis of the protein bands presented in the left panel (the averages of the bands from three clones are presented). Results are presented as mean ± SD (n=3).

Supplementary figure 3

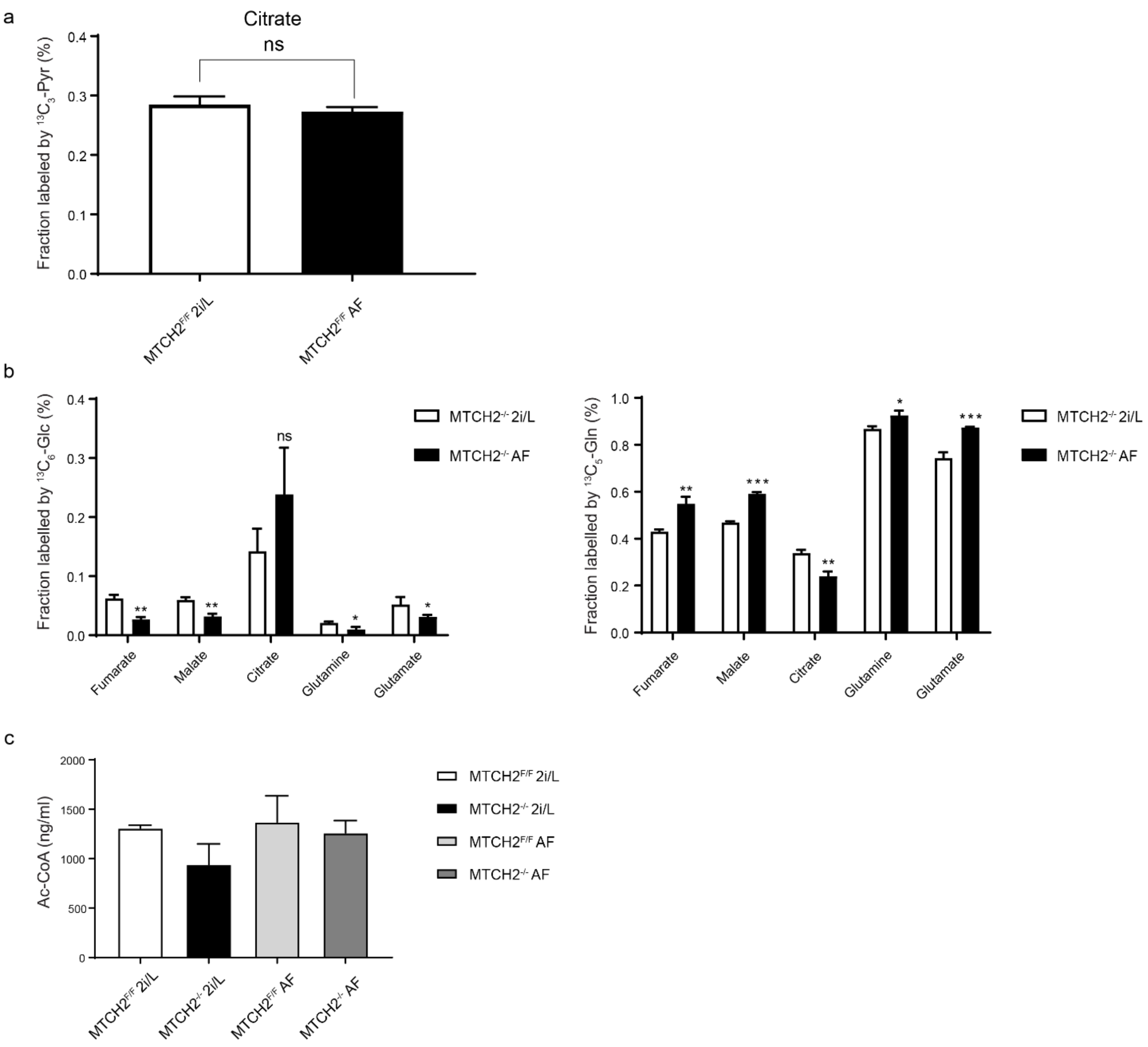

Supplementary figure 3. The effect of MTCH2 loss on several metabolic parameters. (A) No difference in pyruvate oxidation after priming. The fraction of citrate labeled by $^{13}C_3$-pyruvate (M+2) in *MTCH2$^{F/F}$* ESCs and EpiLCs are shown. Results are presented as mean ± SD (n=3). **(B)** Loss of MTCH2 dampens the metabolic shift in the naïve-to-primed transition. Mass isotope tracing of TCA cycle metabolites in *MTCH2$^{-/-}$* ESCs and EpiLCs. Labeled fractions for each metabolite derived from $^{13}C_6$-glucose (^{13}C-glc; M+2 is shown; left panel) or derived from

$^{13}C_5$-glutamine (^{13}C-gln; M+4 or M+5 are shown; right panel) are plotted, after 6 hours incubation with each ^{13}C labeled substrate separately. Percentages were calculated using the Metran software. Results are presented as mean ± SD (*p<0.05; **p<0.01, ***p<0.001; n=3). **(C)** Loss of MTCH2 does not affect the cellular levels of Acetyl-CoA. The Ac-CoA levels in whole cell lysates prepared from *MTCH2*$^{F/F}$ and *MTCH2*$^{-/-}$ ESCs and EpiLCs are presented. No differences were detected between the samples using ANOVA statistical assay. Results are presented as mean ± SD (n=3).

Supplementary figure 4

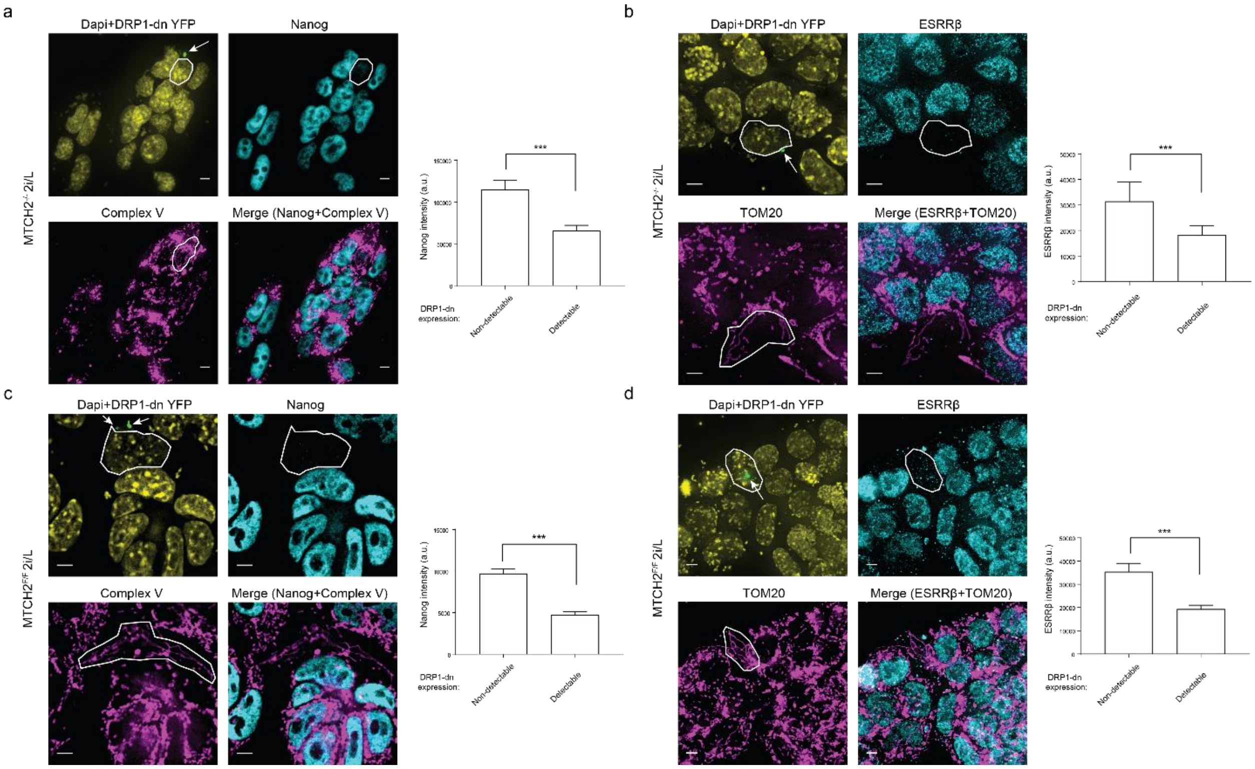

Supplementary figure 4. Mitochondrial elongation by a dominant negative DRP1 drives exit from naïve pluripotency.

(A) Expression of a dominant negative form of the pro-fission protein dynamin-related protein 1 (DRP1-dn YFP) in *MTCH2*$^{-/-}$ ESCs induces mitochondria elongation and represses Nanog expression levels. Left panels: Representative IF images showing Nanog expression in *MTCH2*$^{-/-}$ ESCs expressing DRP1-dn (Scale bar, 5 μm). The cell expressing DRP1-dn YFP and repressing Nanog levels is marked by a white line. The white arrowhead mark YFP foci indicative of a cell expressing DRP1-dn YFP. Right panel: Average nuclear fluorescent intensity of Nanog in *MTCH2*$^{-/-}$ ESCs with either detectable or non-detectable expression of DRP1-dn YFP. Results are presented as mean ± SEM (****p*<0.001; n=17 cells for each group). **(B)** Inducing mitochondrial elongation by expressing DRP1-dn in *MTCH2*$^{-/-}$ ESCs represses ESRRβ expression levels (Scale bar, 5 μm). Left panels: Representative IF images showing ESRRβ expression in *MTCH2*$^{-/-}$ ESCs

expressing DRP1-dn. The cell expressing DRP1-dn YFP and repressing ESRRβ levels is marked by a white line. The white arrowhead marks YFP foci indicative of a cell expressing DRP1-dn YFP. Right panel: Average nuclear fluorescent intensity of ESRRβ in *MTCH2*$^{-/-}$ ESCs with either detectable or non-detectable expression of DRP1-dn YFP. Results are presented as mean ± SEM (***p<0.001; n=13 cells for each group). **(C)** Expression of DRP1-dn YFP in *MTCH2*$^{F/F}$ ESCs induces mitochondria elongation and represses nuclear Nanog expression levels. Left and right panels: As in (A). Results are presented as mean ± SEM (***p<0.001; n=13 cells for each group). **(D)** Inducing mitochondrial elongation by expressing DRP1-dn in *MTCH2*$^{F/F}$ ESCs represses ESRRβ expression levels. . Left and right panels: As in (B). Results are presented as mean ± SEM (***p<0.001; n=14 cells for each group).

Supplementary figure 5

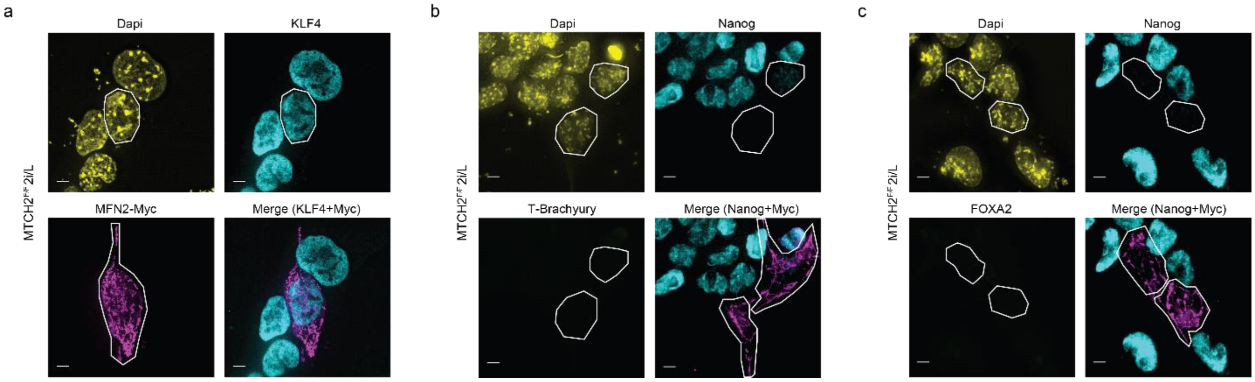

Supplementary figure 5. Enforced Mitochondrial elongation does not repress KLF4 or induce early differentiation markers.

(A) MFN2-Myc induces mitochondrial fusion but does not repress the expression of the naïve marker KLF4 in *MTCH2*$^{F/F}$ ESCs. Representative IF images showing KLF4 expression in *MTCH2* $^{F/F}$ ESCs expressing MFN2-Myc (Scale bar, 5 μm). The cell expressing MFN2-Myc and not repressing KLF4 is marked with a white line. **(B)** MFN2-Myc induces mitochondrial fusion but does not induce the expression of T-brachyury. Representative IF images showing the lack of T-brachyury expression in *MTCH2* $^{F/F}$ ESCs expressing MFN2-Myc and repressing Nanog expression (Scale bar, 5 μm). The cells expressing MFN2-Myc and repressing Nanog expression but not expressing T-brachyury are marked with white lines. **(C)** MFN2-Myc induces mitochondrial fusion but does not induce the expression of FOXA2. Representative IF images showing the lack of FOXA2 expression in *MTCH2* $^{F/F}$ ESCs expressing MFN2-Myc and repressing Nanog expression (Scale bar, 5 μm). The cells expressing MFN2-Myc and repressing Nanog expression but not expressing FOXA2 are marked with white lines.

Supplementary figure 6

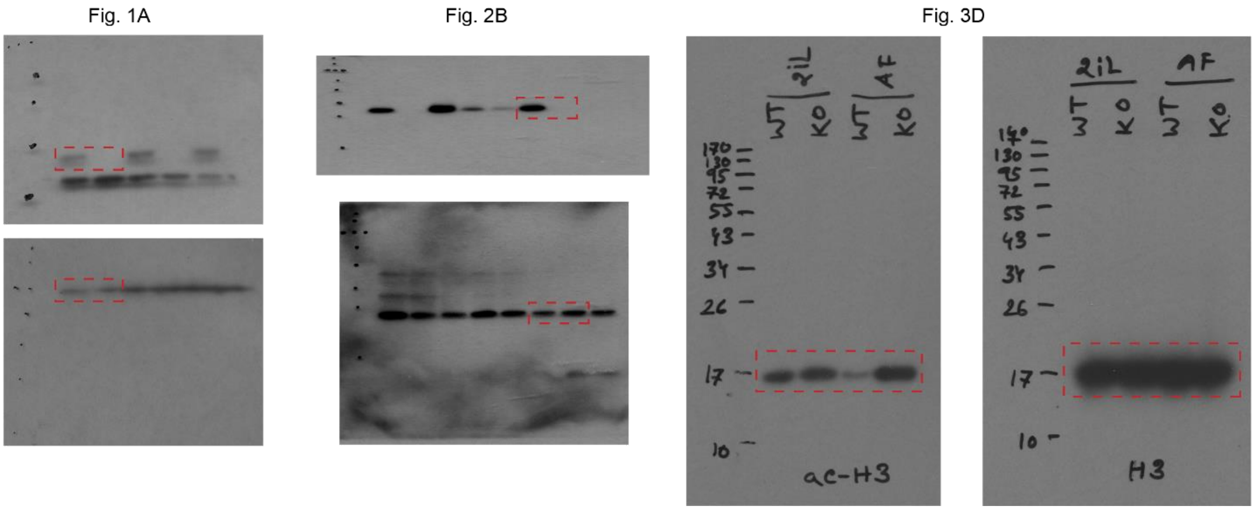

Supplementary figure 6. Full scans of Western blots shown in main text. Cropped areas are marked by red color.
